# Research on the Mechanical Properties and Modification Mechanisms of Orthogonal Optimization Composite Cement-Based Thin Spray On-Liner

**DOI:** 10.3390/ma18081837

**Published:** 2025-04-17

**Authors:** Diantao Zheng, Xinming Chen, Huazhe Jiao, Liuhua Yang, Xiaohui Liu, Yulong Han, Ziyang Liu

**Affiliations:** 1 Henan Key Laboratory of Underground Engineering and Disaster Prevention and Control, Henan Polytechnic University, Jiaozuo 454003, China; 212208020076@hpu.edu.cn (D.Z.); yanglh2005@163.com (L.Y.); 15838667629@163.com (Y.H.); 18339772285@163.com (Z.L.); 2 College of Civil Engineering, Henan Polytechnic University, Jiaozuo 454150, China; 3 School of Safety Engineering, North China Institute of Science and Technology, Langfang 065201, China; 18338091971@163.com

**Keywords:** thin spray-on liner, acrylic emulsions, mechanical properties, modification mechanism, microscopic analysis

## Abstract

Thin spray on-liner (TSL) is a new type of rock support technology, but ordinary cement-based TSL has low tensile strength and poor toughness, which makes it difficult to meet the challenges of large deformation of coal mine roadway perimeter rock surface maintenance. A high-performance composite cement-based TSL was obtained by adding acrylic emulsion, basalt fiber and rubber powder to modify ordinary Portland cement. The orthogonal test and range analysis method were used to systematically study the change law of the physical and mechanical properties of the composite cement-based TSL, determine its reasonable ratio, and further microscopic analysis to find out the modification mechanism. The results show that the reasonable ratio of composite cement-based TSL is as follows: polymer–cement ratio is 1.75, basalt fiber content is 1%, and rubber powder content is 3%; that is, the viscosity is 20,000 mps, and the elongation, tensile strength and adhesive strength in 28 d are 121%, 2.28 Mpa, and 1.66 Mpa, respectively. When the acrylic emulsion-basalt fiber-rubber powder is compositely modified, the acrylic emulsion cures and the cement hydration product to form a three-dimensional space network structure, which increases the compactness, the basalt fiber reduces the porosity of the matrix, inhibits the development of matrix cracks, and the rubber powder improves the elongation of the matrix and jointly improves the mechanical properties of TSL. This study provides a theoretical basis for the preparation of composite cement-based TSL.

## 1. Introduction

In the 1980s, Canada first proposed TSL, which realizes the closure and support of the surrounding rock by spraying a 3–5 mm thin spray material on the surface of the surrounding rock. At first, the TSL was positioned as a closure material, and in the process of application, it was found to have a certain support capacity, which can be used to improve the self-supporting deformation capacity of the surrounding rock, and to control underground geotechnical engineering disasters such as rock explosion and large deformation [1,2].

TSL can be divided into reactive and non-reactive categories, according to their composition [3]. Reactive TSLs are mainly polymer-based materials, with advantages such as high strength, good film-forming properties, controllable condensation time, and high bonding strength, but they have shortcomings such as high cost, toxicity, and easy-to-detonate gas or coal dust when reacting with warming, so their use in coal mines is limited. Non-reactive TSLs are mainly cement-based materials, which have the advantages of low cost and simple construction facilities, but they have the problems of low strength and poor stability. Therefore, it is necessary to modify the traditional cement-based TSL and develop a composite TSL that maintains good toughness, tensile strength, bond strength, etc., but also meets environmental protection and economic requirements.

In view of the above problems, researchers choose various additives to modify cement-based TSLs, among which polymers and fibers are the two most commonly used additives. Tianye [4] used an acrylic emulsion to modify a cement-based TSL, which greatly improved its tensile and bonding properties. M. Stefanidou et al.’s [5] modification of cementitious materials with a renewable wood fiber was found to significantly improve their compatibility. Li [6,7] found that the use of redispersible latex powder, polypropylene fiber, PS emulsion, and other mixed preparation of flexible shotcrete materials can reduce its jet rebound rate, and improve flexibility and crack resistance. Gopi Krishna Dondapat [8] used a glass fiber-reinforced TSL to coat the surface of rock-like materials and coal samples, and found that it can significantly enhance the compressive properties of rock. Qiao Qiao [9] prepared TSL by modifying silicate and sulphoaluminate composite cement with redispersible latex powder, which significantly improved the tensile toughness of TSL, but the setting time increased. Dong [10] used VAE, pure acrylic acid, and styrene-acrylic emulsion for three kinds of polymer modified cement-based TSLs. Through experiments, it was found that polymer enhanced the flexural strength of TSL, but reduced their compressive strength. Shan Z [11] investigated the flexural damage resistance of fiber-reinforced polymer TSL and steel mesh, and found that fiber-reinforced polymer TSLs were not weaker than steel mesh in supporting flexural plates. Nong Zhang [12] investigated the effect of polypropylene fibers on the tensile properties of polymer–cement-based TSL, and the results showed that the incorporation of fibers increased the tensile properties of the materials, while reducing the elongation of the materials. In summary, the use of a single additive can only improve some of the characteristics of TSLs, and it is difficult to take into account multiple properties at the same time. Polymers can improve the tensile properties of cement-based TSLs, but will reduce the compressive strength. Fibers can improve the tensile properties of cement-based TSLs, but have an adverse effect on its elongation. In this paper, ordinary Portland cement is taken as the research object; acrylic emulsion, basalt fiber, and rubber powder are used as additives for composite modification. Orthogonal test and range analysis methods are used to test their physical and mechanical properties and micro morphologies, to explore the influence of acrylic emulsion, basalt fiber, and rubber powder on the performance of composite cement-based TSLs, and to determine the reasonable proportion of composite cement-based TSLs. The research provides a theoretical basis for the preparation of composite cement-based TSLs.

## 2. Materials and Methods

### 2.1. Raw Materials

(1) Cement: P·O 42.5 grade cement produced by China Jiaozuo Qianye Cement Co., Ltd. (Jiaozuo, China). The main chemical composition of cement is shown in Table 1.

(2) Acrylic emulsion: Acrylic emulsion produced by China Guangzhou Kuangxuan Chemical Co., Ltd. (Guangzhou, China). The acrylic emulsion is milky white and liquid, with a solid content of (58 ± 2)% and a viscosity of 500~800 mPa·s.

(3) Rubber powder: The rubber particles used in the test are all made of waste tires by cutting and crushing process. The technical indexes of rubber powder are shown in Table 2.

(4) Basalt fiber: The fiber used in the test is 3–6 mm basalt fiber. The basic mechanical properties of basalt fiber are shown in Table 3.

### 2.2. Test Proportioning Design

In this paper, a single-factor pre-experiment was carried out before the orthogonal test. The mechanical properties were used as indicators to determine the optimal ratio range of single-factor experimental materials: polymer–cement ratio of 1.50–2.00, basalt fiber content of 0.5–1.5%, and rubber powder content of 1–5%. In order to ensure the working performance of the TSL, the amount of defoamer is 1%, and the amount of curing agent is 2%.

Taking ordinary Portland cement as the base material, an orthogonal test was used to explore the influence of polymer–cement ratio (A), basalt fiber content (B), and rubber powder content (C) on the mechanical properties of cement-based TSLs. Each factor took 3 levels, and a total of 9 groups of tests were carried out. The specific test plan is shown in Table 4.

### 2.3. Test Methods

**Figure 1:**
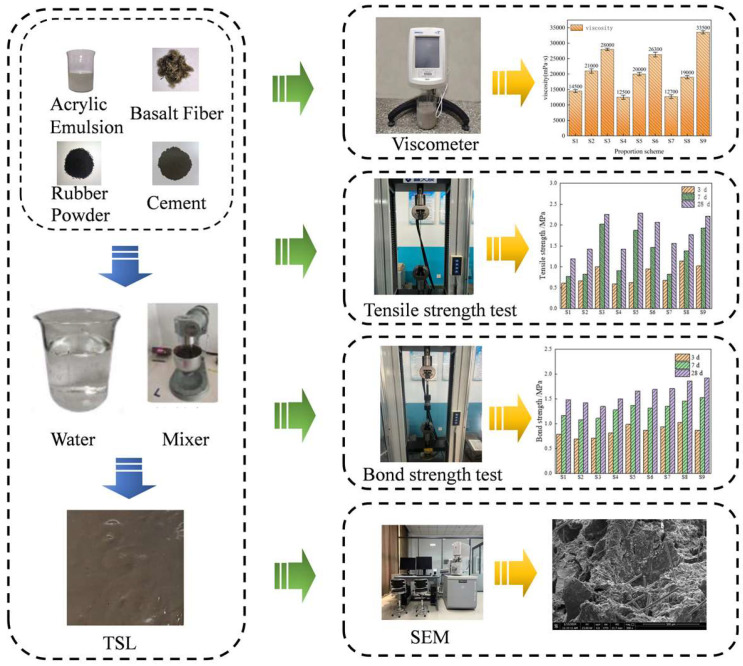
Test scheme.

(1) Viscosity test: Brookfield DV2TLV viscometer (Middleboro, MA, USA) was used to monitor the viscosity of thin spray material in real time. Rotor No.6 was selected to monitor the viscosity of thin spray material in real time. The rotor speed was set to 12.0 RPM. The process was as shown in Figure 1.

(2) Elongation test: the large deformation extensometer was used to measure the deformation of the specimen within the gauge.

(3) Tensile test: ASTM D638 standard was used to test the tensile properties of thin spray materials, and the thickness of the sample was 4 mm. The loading equipment is a microcomputer-controlled electronic universal testing machine, and the loading speed is 20 mm/min.

(4) Bonding test: The original surface core of sandstone with a size of 50 × 50 mm is anchored in the carrier by epoxy resin anchoring agent. Thin spraying material is sprayed on the surface, and epoxy resin anchoring agent is sprayed on the other side. The loading equipment is a microcomputer-controlled electronic universal testing machine, the loading speed is 5 mm/min.

(5) Scanning electron microscope (SEM) test: The conductive layer was formed on the surface of the sample by the Cressington automatic ion sputtering instrument 108AUTO (Watford, UK) to improve the conductivity and stability of the sample. The microstructure was observed by Quanta FEG 250 scanning (Zaragoza, Spain) electron microscope.

## 3. Results and Analysis

### 3.1. Viscosity

**Figure 2:**
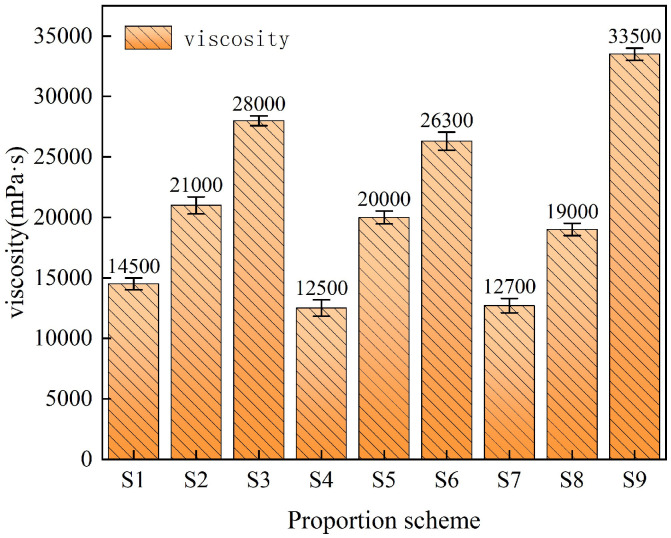
Slurry viscosity of composite TSL with different ratios.

**Figure 3:**
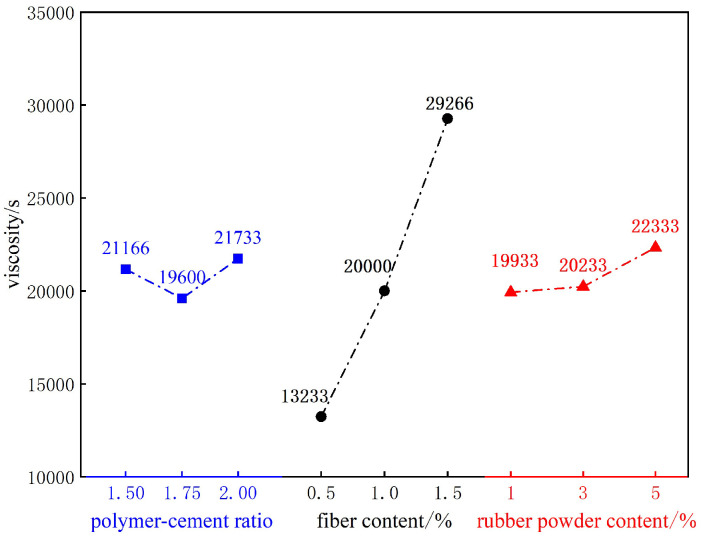
Effects of various factors on viscosity.

Viscosity reflects the viscous resistance of fluid flow. Both excessively high and low viscosity have an impact on the strength and fluidity of thin-spray materials. Therefore, it is very important to control the viscosity to achieve the desired mechanical and working properties [13]. Figure 2 is the viscosity test results of composite TSL with different ratios. According to the data in the figure, the mean and range of the viscosity of each factor at different levels are calculated. The results are shown in Figure 3 and Table 5. It can be seen from Figure 3 that the viscosity of the composite TSL is positively correlated with the content of basalt fiber and rubber powder. When the polymer–cement ratio is less than 1.75, there is a positive correlation, and when it is greater than 1.75, there is a negative correlation. It can be seen from Table 5 that the influence of various factors on the viscosity of the composite TSL is basalt fiber content > rubber powder content > polymer–cement ratio. The effect of polymer–cement ratio on viscosity decreases first, and then increases, and the effect of polymer–cement ratio on viscosity is small. The optimum ratio of viscosity is A3B3C3, the ratio of polymer to cement is 2.0, the content of basalt fiber is 1.5%, and the content of rubber powder is 5.0%.

When the content of acrylic emulsion is less, a large number of surface active substances in the emulsion participate in the flow, and the viscosity decreases. With the increase in acrylic emulsion content, due to its air-entraining effect, the bubbles in the slurry increase, the consistency and viscosity of the TSL slurry increase [14]. Basalt fiber is a dry material with certain water absorption. With the increase in fiber content, the contact area between fiber and cement matrix increases, the water absorption increases, the water–cement ratio of slurry decreases, and the viscosity increases. On the other hand, the basalt fiber is uniformly dispersed in the slurry to form a network structure, resulting in an increase in friction resistance and shear force, thereby increasing the viscosity of the slurry. The surface of rubber powder is uneven, and it will adsorb the mixing water after adding. The water–cement ratio of the slurry decreases, and the viscosity increases [15].

### 3.2. Elongation

**Figure 4:**
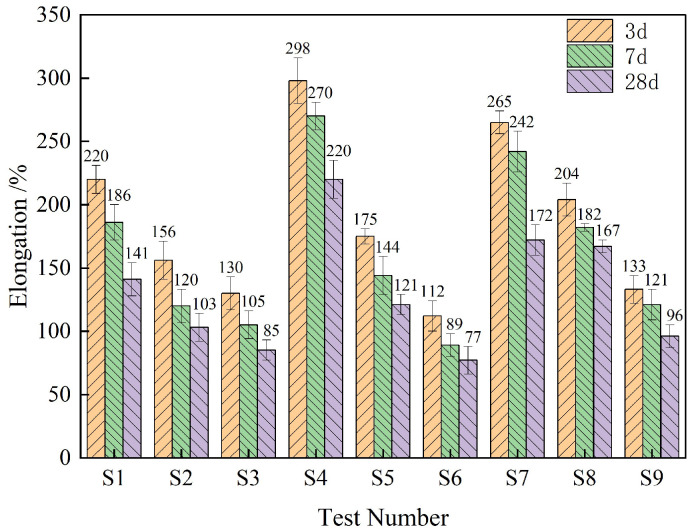
The elongation at different ages of composite TSL with different proportions.

**Figure 5:**
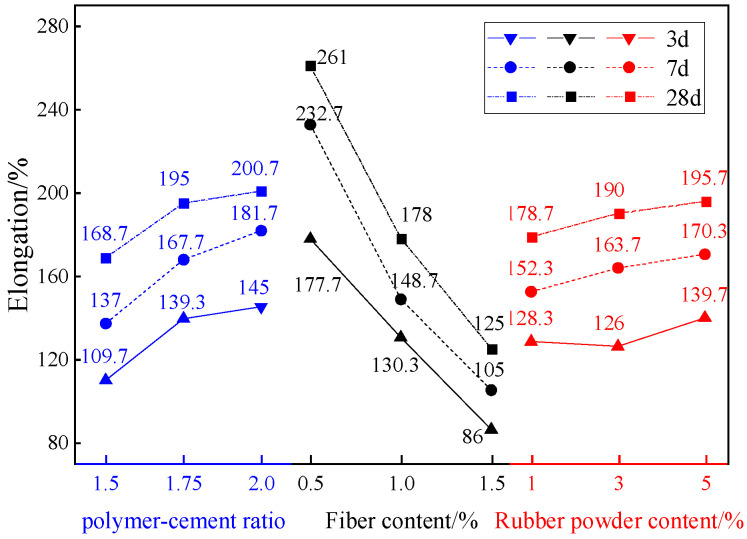
Effects of various factors on the elongation of TSL at different ages.

Figure 4 shows the test results of fracture elongation at different ages of composite thin spray materials with different proportions. According to the data in the figure, the mean and range of tensile strength of TSL at different levels and different ages are calculated respectively. The results are shown in Figure 5 and Table 6.

It can be seen from Figure 4 that the elongation of composite TSL at different ages is roughly positively correlated with the polymer–cement ratio and rubber powder content, and is roughly negatively correlated with the basalt fiber content. It can be seen from Table 6 that the influence of various factors on the composite TSL is basalt fiber > polymer–cement ratio > rubber powder content. Acrylic emulsion and rubber powder increased the elongation, and basalt fiber reduced the elongation. The optimal ratio of elongation is A3B1C3, that is, the polymer cement ratio is 2.0, the basalt fiber content is 0.5%, and the rubber powder content is 5%.

### 3.3. Tensile Strength Analysis

**Figure 6:**
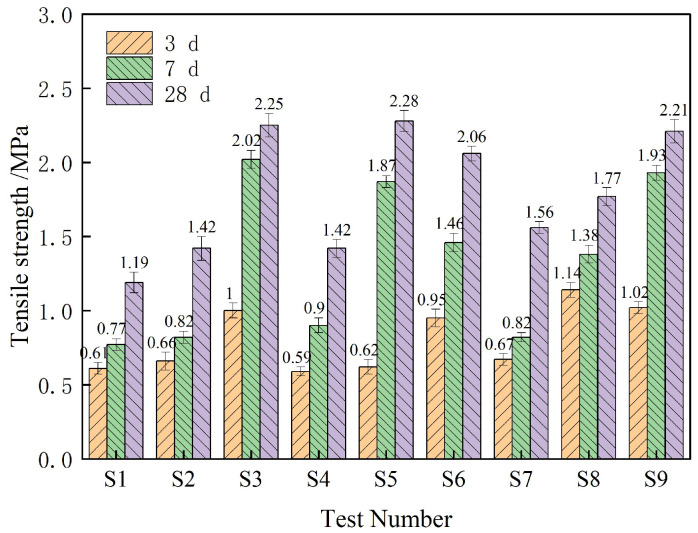
Tensile strength of different proportions of composite TSL at different ages.

**Figure 7:**
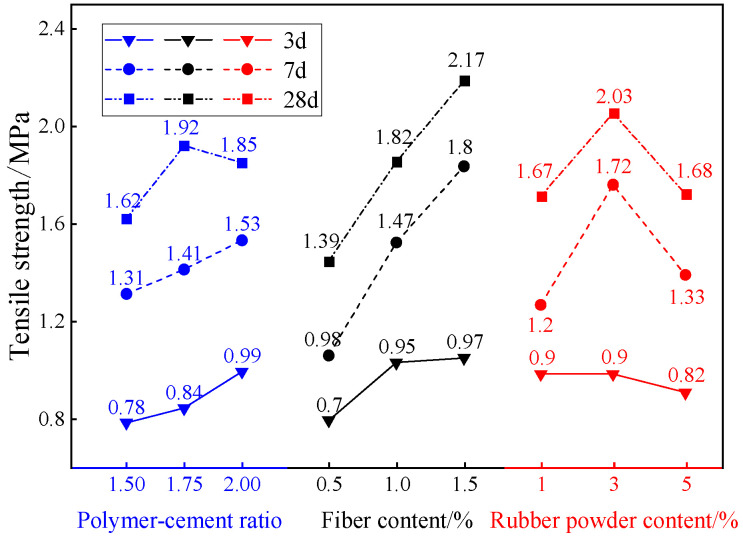
The influence of various factors on the tensile strength of TSL at different ages.

Figure 6 shows the tensile strength test results of different proportions of composite TSL at different ages. According to the data in the figure, the mean and range of the tensile strength of each factor at different levels and different ages are calculated respectively. The results are shown in Figure 7 and Table 7. It can be seen from Figure 7 that the tensile strength of composite TSL at different ages is positively correlated with the polymer–cement ratio and basalt fiber content. When the rubber powder content is less than 3%, there is a positive correlation, and when it is greater than 3%, there is a negative correlation. It can be seen from Table 7 that the basalt fiber has the greatest influence on the tensile strength of the composite TSL, which improves the tensile strength of the TSL. The polymer–cement ratio has a great influence on the 3 d age TSL, and the rubber powder content has a great influence on the 7 d and 28 d TSL, which increases first and then decreases the tensile strength of the TSL. The optimal ratio of the tensile strength of the 28 d TSL is A_2_B_3_C_2_; that is, the polymer–cement ratio is 1.75, the basalt fiber content is 1.5%, and the rubber powder content is 3%.

With the increase in the ratio of polymer to cement, the content of acrylic emulsion increases, and the tensile strength of the composite TSL increases. Since the acrylic emulsion will form a continuous polymer film in the TSL, and the cement and cement hydration products are interwoven to form a space grid structure, covering the microcracks inside the TSL slurry and the surface of the unhydrated cement particles, the integrity of the internal structure is improved, and the tensile strength of the TSL is improved [16,17]. Basalt fiber has high tensile strength and good adhesion with cement in the TSL. Therefore, when the TSL is loaded and reaches the cracking load, the basalt fiber at the crack can effectively transfer the load between the matrix at both ends of the crack, so that the cracked TSL continues to play a role, thereby improving the tensile strength of the TSL [18,19]. The rubber powder plays a role in filling the gap of the TSL, increasing the density of the TSL and improving the tensile strength of the TSL. When the content of rubber powder is more than 3%, it is equivalent to the introduction of organic impurities in the TSL. The interface bonding force between rubber powder and cement and propylene emulsion is poor, and the hydration product with bonding strength cannot be formed with cement, and the tensile strength of the TSL is reduced [20].

### 3.4. Bond Strength Analysis

**Figure 8:**
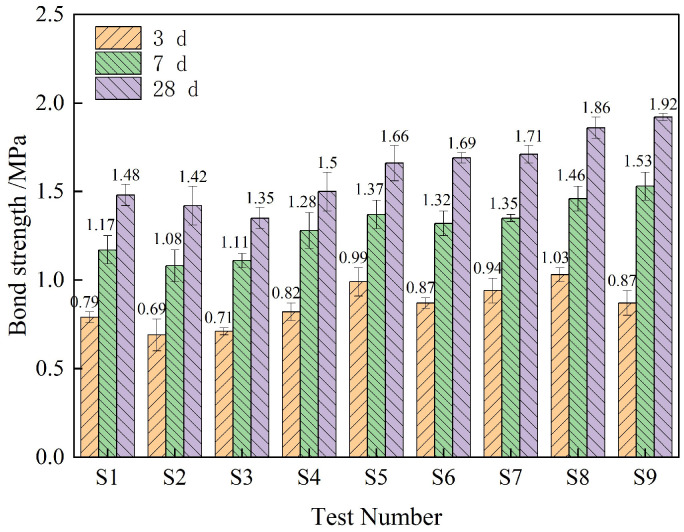
Bond strength of composite TSL with different proportions at different ages.

**Figure 9:**
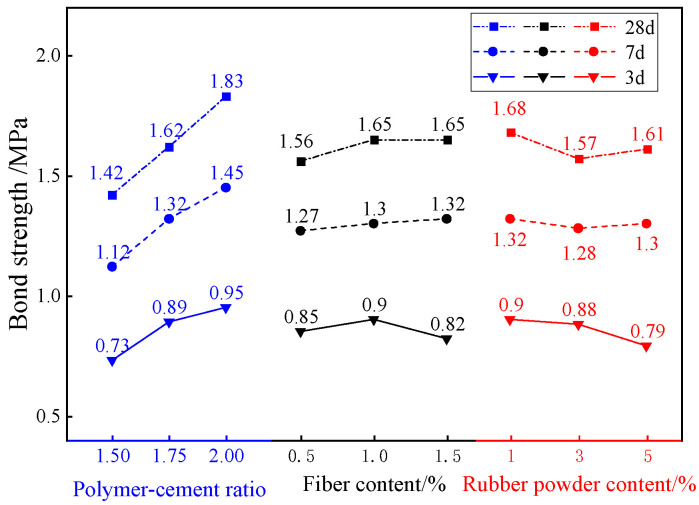
The effects of various factors on the bond strength of composite TSL at different ages.

Figure 8 is the uniaxial tensile bond strength test results of composite TSL with different ratios at different ages. According to the data in the figure, the mean and range of the uniaxial tensile bond strength calculation of each factor at different levels and different ages are calculated respectively. The results are shown in Figure 9 and Table 8. From Figure 9, it can be seen that the uniaxial tensile bond strength of the composite TSL at different ages is positively correlated with the polymer–cement ratio; it is negatively correlated with the amount of rubber powder; there is a positive correlation when the basalt fiber content is less than 3%, and a negative correlation when it is greater than 3%. It can be seen from Table 8 that the polymer–cement ratio has the greatest influence on the bond strength of the TSL, which increases the bond strength of the TSL. The optimal ratio of the bond strength of the 28 d TSL is A_3_B_2_C_1_; that is, the polymer–cement ratio is 2.0, the basalt fiber content is 1.0%, and the rubber powder content is 1%.

As an organic compound, acrylic emulsion is added to TSL to form an interpenetrating network spatial structure, which increases the bonding force with the interface, thereby improving its bonding strength. With the increase in basalt fiber content, the bond strength of composite TSL increases first and then decreases. The basalt fiber is evenly distributed at the bonding interface, which can inhibit the original microcracks generated at the interface during the hydration of the TSL cement, and can effectively inhibit the development of cracks when cracks occur at the interface. However, excessive fibers are prone to agglomeration, and there are large pores at the fiber-bond interface, which increases the internal defects of the TSL and reduces the force transmission of the fiber, resulting in a decrease in bond strength [21].

### 3.5. Performance of Composite TSL with Optimal Ratio

The comprehensive orthogonal test results show that the improvement of the physical and mechanical properties of the composite cement-based TSL is the result of the combined action of acrylic emulsion, basalt fiber, and rubber powder. The appropriate amount of acrylic emulsion will improve the mechanical properties and toughness of the TSL, but will reduce the viscosity; the basalt fiber can improve the tensile strength and bond strength of the TSL, but the content should not be too high; rubber powder can improve the elongation of TSL, but too high a content has adverse effects on mechanical properties. Based on the comprehensive performance and economic cost, the ratio of composite cement-based TSL is determined to be A_2_B_2_C_2_, that is, the polymer–cement ratio is 1.75, the basalt fiber content is 1%, and the rubber powder content is 3%.

## 4. Microstructure and Mechanism Analysis

### 4.1. SEM Micromorphology Analysis

**Figure 10:**
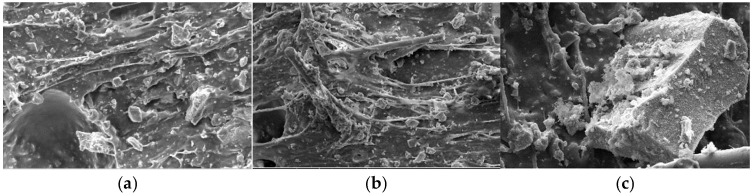
Microstructure of hydration 28 d of composite TSL. (**a**) SEM image of polymer-containing scanned electron microscope; (**b**) SEM image of fiber-containing fibers; (**c**) SEM image with rubber powder.

From the microscopic point of view, the material morphology and pore structure distribution of the sample were observed by scanning electron microscope (SEM) from the fracture site, and the modification mechanism was analyzed. Acrylic emulsion has a certain adsorption and complexation, with its curing dehydration forming a continuous polymer film, closed holes, and micro-cracks, which improve the internal defects and microstructure of the TSL, and the polymer film and cement hydration products form an interpenetrating space network structure improve its mechanical properties [22]. Figure 10 is the micro-morphology of the optimal ratio of composite thin spray material cured for 28 d. From Figure 10a, it can be seen that the cement particles and hydration products are wrapped by the acrylic emulsion solidified polymer film. The polymer film is evenly distributed and wrapped with the cement hydration products to form an interconnected network structure, which improves the interface transition zone and forms a continuous structure. It can be seen from Figure 10b that the basalt fiber is used as a filler in the matrix to bond the pores inside the TSL and reduce its internal porosity. On the other hand, the basalt fiber plays a ‘bridging’ role in the matrix, which restricts the expansion of the matrix crack and enhances the mechanical properties of the composite cement-based TSL. It can be seen from Figure 10c that it is well bonded to the cement product and the polymer film, and the structure is relatively dense.

### 4.2. Mechanism Analysis and Discussion

**Figure 11:**
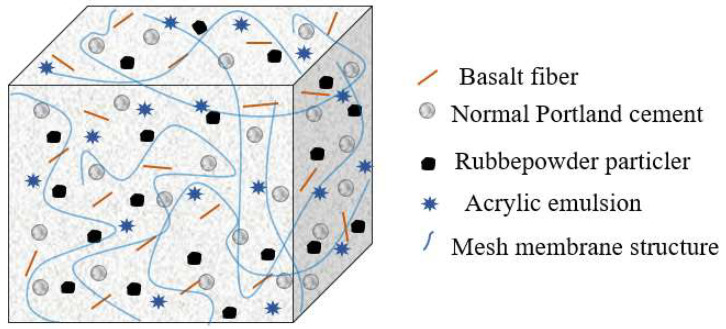
Composition diagram of composite TSL system.

The composite cement-based TSL is a multi-directional system material, and its system composition diagram is shown in Figure 11. At the same time of cement hydration in the TSL, the acrylic emulsion loses water, and the polymer particles flocculate together to form a polymer sealing layer on the surface of the cement hydration gel. Due to the continuous hydration process, the water between the condensed polymer particles is gradually absorbed into the chemically bound water of the cement hydration process. Finally, the polymer particles and the cement hydrates are completely condensed to form an interconnected network structure. The incorporation of acrylic emulsion replaces part of the cement, and the elastic modulus of the polymer film is low, which improves the stress state inside the thin spray material. It can withstand deformation to reduce the stress of the cement stone, and the possibility of micro cracks is also reduced accordingly. At the same time, the incorporation of basalt fiber plays a role in bridging and filling, and the fiber inhibits the spread of cracks.

## 5. Results and Discussion

This study examines the impact of incorporating acrylic emulsion, basalt fiber, and rubber powder on the physical and mechanical properties of cementitious composite thin spray materials. The primary findings of this research are as follows.

(1) The orthogonal test results show that the reasonable mix ratio of composite cement-based TSL is polymer–cement ratio 1.75, basalt fiber content 1%, rubber powder content 3%. The tensile strength of the modified composite cementitious TSL reached 2.28 MPa, and the bond strength reached 1.66 MPa. Compared with the ordinary cementitious TSL, the mechanical properties were significantly improved.

(2) The modification mechanism of acrylic emulsion, basalt fiber and rubber powder was analyzed by analyzing the SEM microstructure of composite cement-based TSL. The acrylic emulsion fills the pores of the cementitious material, forms a three-dimensional space network structure with the cement paste, and improves the mechanical properties of the TSL. An appropriate amount of basalt fiber plays a ‘bridging‘ role in the TSL and restricts the expansion of the matrix crack. The elastic modulus of rubber powder is small, and the polymer film forms an interwoven spatial network structure with cement hydration products and rubber powder, which improves the toughness of TSL.

In summary, the use of acrylic emulsion, basalt fiber, and rubber powder as modifiers can solve the problems of low tensile strength and poor toughness of traditional cement-based TSL, thus improving their mechanical properties. This provides a feasible way for high flexibility and toughening modification of cement-based TSL. However, in order to further improve the mechanical properties of TSL, it is necessary to further explore other additives to further optimize the mechanical properties of TSL.

## Figures and Tables

**Table 1:** Main chemical composition of cement.

Chemical Composition	SiO_2_	AL_2_O_3_	CaO	Fe_2_O_3_	MgO	Other
Percentage (%)	20.61	3.98	65.70	2.62	1.56	5.53

**Table 2:** Rubber powder technical indicators.

Particle Size /mm	Iron Content /%	Fiber Content /%	Sieve Residue /%	Fracture Strength /MPa	Breaking Elongation /%
3–6	0.02	0.00	0.021	16.4	582

**Table 3:** Basic physical and mechanical properties parameters of basalt fiber.

Fiber Category	Fiber Diameter /mm	Density /g·cm^3^	Breaking Strength /MPa	Elastic Modulus /GPa	Breaking Elongation /%
basalt fiber	17.0	2.705	1.71 × 10^3^	75.4	2.5

**Table 4:** TSL orthogonal test material ratio.

Test Number	A (Polymer–Cement Ratio)	B (Basalt Fiber Content)/%	C (Rubber Powder Content)/%
S 1	1.50	0.5	1.0
S 2	1.50	1.0	5.0
S 3	1.50	1.5	3.0
S 4	1.75	0.5	5.0
S 5	1.75	1.0	3.0
S 6	1.75	1.5	1.0
S 7	2.00	0.5	3.0
S 8	2.00	1.0	1.0
S 9	2.00	1.5	5.0

**Table 5:** Viscosity range analysis.

Factor	A	B	C
Extreme difference/mm	2133.33	16,033.33	2400.00
primary and secondary factor	Fiber content > rubber powder viscosity > polymer–cement ratio		
optimal proportion	A3	B3	C3

**Table 6:** Range analysis of elongation of composite TSL at different ages.

Factor	A	B	C
3 d extreme difference/%	32	136	17
The primary and secondary factors of 3 d	Basalt fiber content > polymer–cement ratio > rubber powder content		
The optimal ratio of 3 d	A_3_	B_1_	C_3_
7 d extreme difference/%	44.67	127.67	18
The primary and secondary factors of 7 d	Basalt fiber content > polymer–cement ratio > rubber powder content		
The optimal ratio of 7 d	A_3_	B_1_	C_3_
28 d extreme difference/%	35.33	91.67	13.67
The primary and secondary factors of 28 d	Basalt fiber content > polymer–cement ratio > rubber powder content		
The optimal ratio of 28 d	A_3_	B_1_	C_3_

**Table 7:** Analysis of tensile strength range of composite TSL at different ages.

Factor	A	B	C
3 d extreme difference/%	0.21	0.28	0.08
The primary and secondary factors of 3 d	Basalt fiber content > polymer–cement ratio > rubber powder content		
The optimal ratio of 3 d	A_3_	B_3_	C_1_
7 d extreme difference/%	0.22	0.82	0.52
The primary and secondary factors of 7 d	Basalt fiber content > rubber powder content > polymer–cement ratio		
The optimal ratio of 7 d	A_3_	B_3_	C_2_
28 d extreme difference/%	0.30	0.78	0.36
The primary and secondary factors of 28 d	Basalt fiber content > rubber powder content > polymer–cement ratio		
The optimal ratio of 28 d	A_2_	B_3_	C_2_

**Table 8:** Range analysis of bond strength of composite TSL at different ages.

Factor	A	B	C
3 d extreme difference/%	0.10	0.08	0.15
The primary and secondary factors of 3 d	rubber powder content > polymer–cement ratio > Basalt fiber content		
The optimal ratio of 3 d	2	3	2
7 d extreme difference/%	0.46	0.15	0.06
The primary and secondary factors of 7 d	polymer–cement ratio > Basalt fiber content > rubber powder content		
The optimal ratio of 7 d	3	3	2
28 d extreme difference/%	0.48	0.14	0.04
The primary and secondary factors of 28 d	polymer–cement ratio > Basalt fiber content > rubber powder content		
The optimal ratio of 28 d	3	3	2

## Data Availability

The original contributions presented in the study are included in the article, further inquiries can be directed to the corresponding author.
